# Fever during pregnancy as a risk factor for neurodevelopmental disorders: results from a systematic review and meta-analysis

**DOI:** 10.1186/s13229-021-00464-4

**Published:** 2021-09-18

**Authors:** Stephanie Antoun, Pierre Ellul, Hugo Peyre, Michelle Rosenzwajg, Pierre Gressens, David Klatzmann, Richard Delorme

**Affiliations:** 1 Child and Adolescent Psychiatry Department, Robert Debré Hospital, APHP, Paris University, Paris, France; 2 Immunology-Immunopathology-Immunotherapy (i3), INSERM U959, Sorbonne University, Paris, France; 3 Université de Paris, Inserm UMR, 1141 NeuroDiderot, Paris, France; 4 Centre for the Developing Brain, Division of Imaging Sciences and Biomedical Engineering, King’s College London, King’s Health Partners, St. Thomas’ Hospital, London, UK; 5 Human Genetics and Cognitive Functions, Institut Pasteur, Paris, France

**Keywords:** Maternal immune activation, Autism, Immunology, Children

## Abstract

**Background:**

Fever during pregnancy is a relatively common and most often trivial event. However, under specific conditions, it could affect significantly fetal brain development. Few studies, with inconsistent results, investigated whether fever, regardless the pathogen, could represent a risk factor for neurodevelopmental disorders (NDD) in the offspring. We aimed to explore further this question by performing a systematic review and meta-analysis.

**Methods:**

Peer-reviewed studies exploring the occurrence of NDD in offspring after a fetal exposure to maternal fever were included. We specifically considered the impact of fever severity and duration, taking into consideration some confounding variables such as the use of antipyretic during pregnancy, the trimester in which the fever arose, the maternal age or smoking at time of gestation. MEDLINE, EMBASE, PsycINFO, Cochrane and Web of Science were searched without language restriction. PRISMA recommendations were followed. Odds ratio (OR) were pooled using random-effects meta-analysis. Heterogeneity in effect size across studies was studied using random-effects meta-regression analysis. (PROSPERO CRD42020182801).

**Results:**

We finally considered ten studies gathering a total of 10,304 children with NDD. Among them, 1394 were exposed to fever during pregnancy. The selected studies were divided into 5 case–control studies and 5 cohort studies. Maternal exposure to fever during pregnancy increased the risk of NDD in offspring with an OR of 1.24 [95% CI: 1.12–1.38]. Secondary analysis revealed an increased risk for NDD when fever occurred during the first trimester of gestation [OR 1.13–95% CI: 1.02–1.26].

**Limitations:**

We excluded studies that considered infections with no evidence of fever. Another potential limitation may be the possible heterogeneity between study designs (cohorts and case–control).

**Conclusion:**

Additional evidence supported the association between fever during pregnancy and increased risk for NDD in offspring. Careful monitoring should be considered for children born from mothers with a febrile episode during pregnancy (specifically during the first trimester).

**Supplementary Information:**

The online version contains supplementary material available at 10.1186/s13229-021-00464-4.

## Background

Neurodevelopmental disorders (NDD) are a heterogeneous group of illnesses that begin during a child’s early brain development [1] and range from specific learning difficulties to severe cognitive impairments [1]. The determinism of NDD remains unclear but depends on the interaction of genetic vulnerability and environmental insults [2, 3]. Twin studies upheld this gene-environment interaction model reporting up to 90% concordance rate in monozygotic twins and 50% in dizygotic [1, 4]. Some of the brain Magnetic Resonance Imaging (MRI) abnormalities reported in children with NDD might suggest a prenatal pathogenesis [5, 6]. Therefore, many authors investigated the role of environmental stressors occurring during the pregnancy, with a specific focus on infections or fever [7].

Fever during pregnancy is common, with approximately 20% of women reporting at least one febrile episode [8, 9]. Despite being a relatively benign condition, fever was associated with several neonatal affections and adverse health affections in offspring, leading sometimes to a significant referral to intensive care [10]. Added to this, evidence from animal studies supplemented the suspicions of fever-induced neurotoxicity, independently from the direct impact of infectious processes [11, 12]. The effect of maternal fever during pregnancy may also explain by itself the increased prevalence of NDD observed in the offspring [3, 13, 14]. This association was suggested to occur secondarily to maternal immune activation (MIA), rather than the direct effect of the pathogen on the fetal brain [14, 15]. In animal studies, MIA during gestation affected the brain development and resulted in behavioral abnormalities, mimicking those from NDD in humans [3, 16, 17]. MIA in rodents and monkeys resulted in socio-communication deficit and stereotyped behaviors in pups similar to the cardinal symptoms of autism spectrum disorder (ASD) in children [18]. Consequently, theories have been raised concerning the detrimental impact of maternal inflammatory balance disruption on the fetal brain development [3]. Maternal pro-inflammatory cytokine interleukin-6 (Il-6) elevation or interleukin-10 (Il-10) decrease, both observed during MIA, might have a key role in yielding abnormal neurodevelopment in offspring [17, 19]. While current evidence did not bring out a direct causal link, genetic susceptibility probably intervened also in the subsequent NDD-related symptoms [3].

However, the literature on fever during pregnancy remains vague on factors modulating the risk of developing a NDD in offspring. For example, the period of pregnancy during which fetal vulnerability is greatest is poorly known. Based on animal models of MIA and taking into account the development of the fetal brain in utero, we assumed that the end of the first trimester and the beginning of the second trimester of pregnancy could represent the period of greatest risk [6, 20–22]. Nevertheless, the neurogenesis begins in the early weeks of pregnancy and the first trimester may also represent an important time window for the fetal risk. This period corresponds to an active period of division and migration of neurons [23]. The use of antipyretic medications during gestation might also be NDD risk factor for offspring, by counteracting the protective effect of anti-inflammatory cytokines. For example, some studies suggested that acetaminophen use during pregnancy increased the risk of Attention-Deficit/Hyperactivity Disorder (ADHD) [24, 25]. Contrariwise, others showed that antipyretics would have a protective effect on NDD emergence in offspring [21, 26–28].

Herein, we present the results of a systematic review and meta-analysis of the literature which explored the association between prenatal exposure to fever, no matter the cause, and NDD outcomes in the offspring.

## Methods

### Search strategy and selection criteria

We performed a systematic review and meta-analysis following the PRISMA recommendations (Preferred Reporting Items for Systematic review and Meta-Analysis) [29]. Studies were included in our systematic review if they met the following criteria: (1) reported the occurrence of any type of NDD that corresponds to DSM-5 criteria; (2) mentioned the history of fever during pregnancy; (3) included a control group that was not diagnosed with any type of NDD (other non-NDD psychiatric controls were not eligible); (4) were with sufficient sample size to allow computation of an odds ratio (OR). Exclusion criteria were: (1) studies assessing only symptoms of NDD but without formal diagnosis; (2) children screened before age 36 months (no formal diagnosis); (3) the sample overlapping (and being smaller) with another eligible study i.e. if studies used the same cohort, we only included the publication reporting the largest number of participants to avoid duplication of data; (4) animal studies.

We searched MEDLINE (1946 to July, 2020), EMBASE (1974 to July, 2020), PsycINFO (1806 to July, 2020), Cochrane Central Register of Controlled Trials (CENTRAL; from inception to July, 2020), and Web of Science Core Collection (1900 to July, 2020) without any restrictions on language, ethnic origins, date or article type. Both cohort and case–control studies were included. The research was based on a combination of medical subject heading (Table 1). We also searched the references in the included studies for any potential pertinent study not detected in the search of databases [29].

**Table 1:** List of medical subject heading

(Fever [tiab] OR Hyperthermia [tiab])
AND
(Pregnancy [tiab] OR Pregnant [tiab] OR in utero [tiab])
AND
(Intellectual Disabilities [tiab] OR Intellectual Developmental Disorder [tiab] OR mental retardation [tiab] OR Global Developmental Delay [tiab] OR Communication Disorders [tiab] OR Language Disorder [tiab] OR Speech Sound Disorder [tiab] OR Childhood-Onset Fluency Disorder [tiab] OR Social Communication Disorder [tiab] OR Pragmatic Communication Disorder [tiab] OR Autism spectrum disorder [tiab] OR Autism [tiab] OR Asperger syndrome [tiab] OR Asperger [tiab] OR ASD [tiab] OR autistic disorder [tiab] OR autistic [tiab] OR attention deficit hyperactivity disorder [tiab] OR adhd [tiab] OR Attention Deficit Disorder with Hyperactivity [tiab] OR Hyperkinetic Disorder [tiab] OR Hyperactivity disorder [tiab] OR hyperactive child syndrome [tiab] OR Attention Deficit Disorder [tiab] OR ADD [tiab] OR attention deficit [tiab] OR minimal brain dysfunction [tiab] OR Specific Learning Disorder [tiab] OR Developmental Coordination Disorder [tiab] OR Stereotypic Movement Disorder [tiab] OR Tic [tiab] OR Tic Disorders [tiab] OR Tourette's disorders [tiab] OR Neurodevelopment [tiab] OR Neurodevelopmental Disorder [tiab])

The eligibility process was conducted in two separate stages: (1) two researchers (SA and PE) independently screened all non-duplicated references initially retrieved as potentially pertinent and excluded those clearly not pertinent based on title or abstract. A final list was agreed with discrepancies resolved by consensus between the two authors. When consensus was not reached, a third, senior researcher (RD) acted as arbitrator; (2) the full-text version of the articles passing stage 1 screening was downloaded and assessed for eligibility by the two researchers, independently. Discrepancies were resolved by consensus between the two researchers and, if needed, the third senior researcher also acted as arbitrator. When required, corresponding authors were contacted to clarify study eligibility.

The protocol for the present systematic review/meta-analysis was pre-registered on the international Prospective Register of Systematic Reviews PROSPERO (protocol number: PROSPERO CRD42020182801).

### Data analysis and outcomes

The main outcome measure was the OR expressing the association between fever in mothers during pregnancy and NDD in the offspring. To further estimate the risk of NDD in the offspring, we performed secondary outcome analysis by taking into account: (1) the types of NDD and the criteria used for their definition; (2) the fever related information regarding the trimester in which it occurred, the prescription of antipyretic medication, and its duration (in days) and the highest fever reached during pregnancy; (3) the offspring’s related main confounding variables including age, sex, week gestational age (WGA) at delivery; and (4) the mothers’ related information: age at conception, smoking during pregnancy and personal history of psychiatric disorders. In addition to these variables, we extracted some information regarding authors, year of publication, country in which the study was conducted.

To assess the quality of studies included in the meta-analysis, SA and PE used a modified version of the Newcastle Ottawa Scale (NOS) [30, 31]. Briefly, the NOS provides assessment criteria for case–control, cross-sectional and cohort studies. Three methodological domains were assessed: the selection criteria, the comparability and the measurement of outcome/exposure. Scoring criteria were amended such that the maximum score available for each study was 8 (Tables 2 and 3). Studies were of high quality if the NOS score was strictly > 4.

**Table 2:** The Newcastle–Ottawa scale scores of the included cohort studies

Study	Representativeness of the exposed cohort	Selection of the non-exposed cohort	Ascertainment of the exposure	Demonstration that outcome of interest was not present at the start of study	Comparability	Assessment of outcome	Follow-up long enough for outcome to occur	Adequacy of follow-up of cohorts	Total
Atladóttir et al. (2012) [14]	1	1	0	1	1	1	1	0	6
Dreier et al. (2016) [6]	1	1	0	1	1	1	1	0	6
Holst et al. (2015) [47]	1	1	0	1	1	1	1	0	6
Gustavson et al. (2019) [48]	1	1	0	1	1	1	1	0	6
Hornig et al. (2018) [21]	1	1	0	1	1	1	1	0	6

**Table 3:** The Newcastle–Ottawa scale scores of the included case–control studies

Study	Case definition	Representativeness of the cases	Selection of controls	Definition of controls	Comparability	Assessment of exposure	Same method of ascertainment	Non-response rate	Total
Brucato et al. (2017) [46]	1	1	1	1	1	0	1	0	6
Christian et al. (2018) [45]	1	1	1	1	1	0	1	1	7
Croen et al. (2019) [22]	1	1	1	0	1	0	1	1	6
Saunders et al. (2019) [44]	1	1	1	0	0	0	1	0	4
Zerbo et al. (2013) [27]	1	1	1	0	1	0	1	0	5

We used a random-effect meta-analysis model. Heterogeneity was assessed using the I^2^ statistic and Tau^2^. The I^2^ score represents the percentage of variation across studies due to heterogeneity, and ranges from zero to 100%; higher values indicate greater heterogeneity across studies [32]. Tau^2^ estimates the between study variance in a random-effect meta-analysis. A Tau^2^ value greater than 1 suggests the presence of substantial heterogeneity [33–35]. The statistical analysis was performed using the R package “meta” for unadjusted OR and “metaphor” for adjusted OR (log-transformed). Meta-analysis of OR was done only when three or more studies were available. To assess publication bias, we used funnel plots. We also performed metaregression on the following confounding factors: impact of fever duration, antipyretic medication, and maternal characteristics. Metaregression aims to examine the impact of the confounding factors on the study. Additional statistical analyses were carried out to stratify by NDD type and by study design, followed by cross stratification.

### Role of the funding source

The funders of the study had no role in study design, data collection, data analysis, data interpretation, or writing of the report. The corresponding author had full access to all the data in the study and had final responsibility for the decision to submit for publication.

## Results

### Literature search and selection

**Fig. 1:**
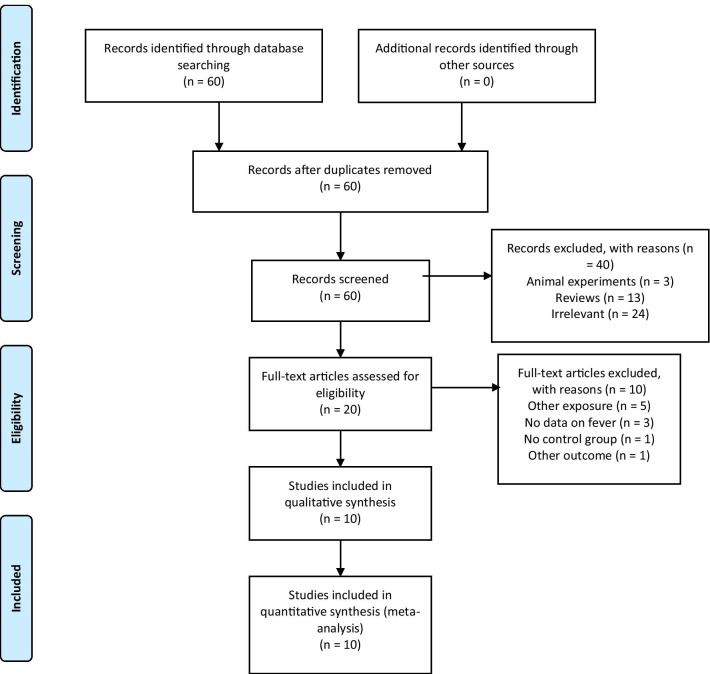
Flowchart of the study inclusion and exclusion process

An initial pool of 60 articles was identified through database searching (MEDLINE, EMBASE, PsyINFO, Cochrane Central Register of Controlled Trials and Web of Science Core Collection). In all, 3 animal experiments, 13 reviews and 24 irrelevant studies were excluded. The eligibility of 20 studies were assessed by full-text review. Of the latter, 10 studies were subsequently excluded [24, 25, 36–43]: five had other primary exposures (acetaminophen use, infection without information about fever, premature birth), three lacked data on fever, one was without a control group, and one assessed the impact on academic performance instead of the occurrence of NDD (Table 4). Ten remaining articles [6, 14, 21, 22, 27, 44–48] met the eligibility criteria for inclusion (Fig. 1).

**Table 4:** Discarded studies with reasons

Author	Reason
Al-Dawood and Albar (1993) [36]	No raw data on fever
Dreier et al. (2017) [37]	Study assessed academic performance, no data on neurodevelopmental disorders
Joseph et al. (2017) [38]	Primary exposure is premature birth
Liew et al. (2014) [24]	Primary exposure is Acetaminophen use
Liew et al. (2016) [39]	Primary exposure is Acetaminophen use
Mattson et al. (2003) [40]	Primary exposure is Varicella during pregnancy
Mazina et al. (2015) [41]	No control group for ASD
Pineda et al. (2003) [42]	No data on fever
Ystrom et al. (2017) [25]	Primary exposure is Acetaminophen use, no data on fever
Zerbo et al. (2017) [43]	No data on fever

### Characteristics of the included studies

The selected studies were published between 2012 and 2019. Five were case–control and five were cohort studies, with a total of 416,446 participants (cohort studies: 411,151; case–control studies: 5295). Among them, 81,124 women were with fever during pregnancy (cohort studies: 80,369; case–control studies: 755). Finally, 10,304 of the offspring were diagnosed with a NDD (cohort studies: 7620; case–control studies: 2684). Among them, 3224 were with a diagnosis of ASD (cohort studies: 1559; case–control studies: 1665), 5156 with a diagnosis of ADHD (only in cohort studies), 905 with a diagnosis of Developmental Coordination Disorder (DCD) (only in cohort studies), and 1019 with a diagnosis of Developmental Delay (DD) (only in case control studies). It is important to note that the cohort studies are from two study populations, namely the Danish National Birth Cohort (three studies) [6, 14, 47] and the Norwegian Mother and Child Cohort Study (two studies) [21, 48]. Characteristics of the selected studies were reported in Table 5.

**Table 5:** Characteristics of the studies of maternal fever during pregnancy and risk of NDD in the offspring

Authors	Year	Location	Study type	NDD	Diagnostic Instrument	Age range (months)	Total participants	Total exposure	Total non-exposure	Total NDD	Total non-NDD	Exposure sex ratio (M/F)	NDD sex ratio (M/F)
Atladóttir et al. [14]	2012	Denmark	Cohort	ASD	ICD10	96–168	96,736	23,128	61,482	976	95,760	–	792:184 (4.30)
Dreier et al. [6]	2016	Denmark	Cohort	ADHD	ICD10 DSM-IV	48–127.2	89,146	24,531	64,426	2215	86,742	–	–
Holst et al. [47]	2015	Denmark	Cohort	DCD	DCDQ07	84–107	29,568	7909	21,603	905	28,607	4078:3887 (1.05)	–
Gustavson et al. [48]	2019	Norway	Cohort	ADHD	ICD10 ADHD-RS	88.8–207.6	99,947	9100	74,572	2941	81,270	4604:4496 (1.02)	–
Hornig et al. [21]	2018	Norway	Cohort	ASD	ICD10 DSM-IV	67.2–182.4	95,754	15,701	80,053	583	95,171	8030:7671 (1.05)	487:96 (5.07)
Saunders et al. [44]	2019	Canada	Case control	ASD	–	5–120	215	31	184	107	108	–	79:28 (2.82)
Croen et al. [22]	2019	USA	Case control	ASD	ADI-R ADOS-2	24–60	2258	367	1891	606	796	–	496:110 (4.51)
				DD	MSEL	24–60				856		–	569:287 (1.98)
Zerbo et al. [27]	2013	USA	Case Control	ASD	ADI-R ADOS-2	24–60	1122	191	931	538	421	–	459:79 (5.81)
				DD	MSEL	24–60				163		–	106:57 (1.86)
Christian et al. [45]	2018	Jamaica	Case Control	ASD	ADI-R ADOS-2	24–96	596	73	523	298	298	–	246:52 (4.73)
Brucato et al. yy[46]	2017	USA	Case Control	ASD	ICD9	–	1104	93	1011	116	988	–	85:31 (2.74)

NDD: Neurodevelopmental Disorder; ASD: Autism Spectrum Disorder; ADHD: Attention Deficit/Hyperactivity Disorder; DCD: Developmental Coordination Disorder; DD: Developmental Delay; ICD10: International Classification of Diseases, tenth revision; DSM-IV: Diagnostic and Statistical Manual of Mental Disorders, fourth edition; DCDQ07: Developmental Coordination Disorder Questionnaire; ADHD-RS: Attention Deficit/Hyperactivity Disorder Rating Scale; ADI-R: Autism Diagnostic Interview-Revised; ADOS-2: Autism Diagnostic Observation Schedule, second edition; MSEL: Mullen Scales of Early Learning; ICD9: International Classification of Diseases, ninth revision

### Maternal exposure to fever during pregnancy and risk of NDD in offspring

**Fig. 2:**
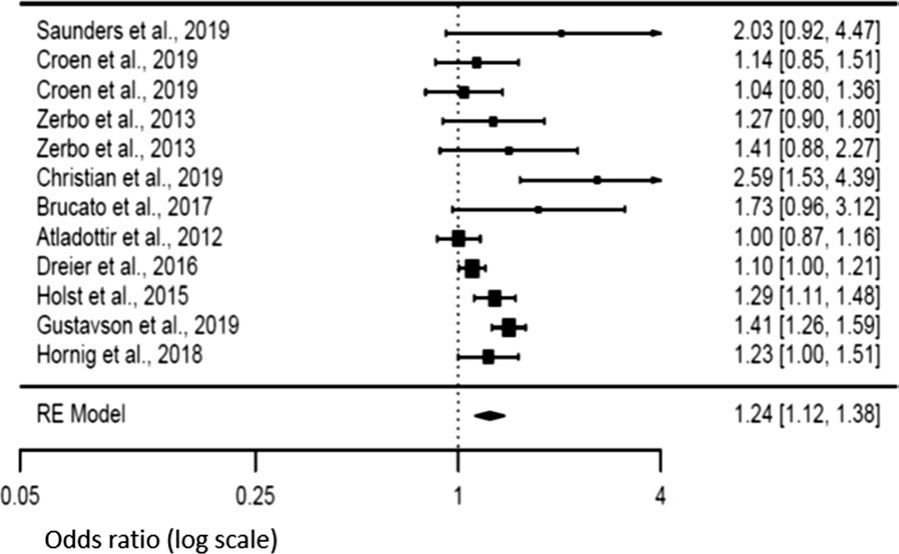
Forest plot relative to maternal fever during pregnancy

A significant association was found between fever during pregnancy and the risk of NDD in offspring (*p*-value < 0.0001), with a pooled odds ratio (OR) of 1.24 [95% confidence interval (CI) 1.12–1.38] (Fig. 2). There was however evidence of heterogeneity [Tau^2^ = 0.016, degrees of freedom (*df*) = 11, *p* < 0.001, *I*^2^ = 61.02] and we observed a funnel plot asymmetry (Additional file 1: Supplemental figures). The group disparity appeared as the result of small sample sizes in some of the selected studies.

### Stratification analyses

Additional statistical analyses were carried out to stratify by NDD type. There was a positive association for ASD (*p* = 0.03), with an OR of 1.15 [95% CI 1.01–1.31], and a low evidence of heterogeneity [Tau^2^ = 0.0076, *df* = 6, *p* = 0.24, *I*^2^ = 24.9%]. We also observed a positive association for DD (*p* = 0.03) with an OR of 1.23 [95% CI 1.07–1.41] and low heterogeneity [Tau^2^ = 0.0019, *df* = 2, *p* = 0.33, *I*^2^ = 9.7%]. There was no possibility to calculate the OR for ADHD, with only two studies, nor for DCD, with only one study.

Stratification by type of study design were also carried out. We found a positive association (*p* = 0.01) for case control studies taken alone with an OR of 1.19 [95% CI 1.03–1.38], with no evidence of heterogeneity [Tau^2^ = 0, *df* = 6, *p* = 0.46, *I*^2^ = 0%]. There was also a positive association for cohort studies (*p* = 0.004) with an OR of 1.20 [95% CI 1.06–1.36], with high evidence of heterogeneity [Tau^2^ = 0.0152, *df* = 4, *p* = 0.0015, *I*^2^ = 77.3%].

We carried out cross stratification between study design and specific NDD type. Only ASD in case control studies were taken into account due to the lack of data. We found a positive association (*p* = 0.02) with an OR of 1.24 [95% CI 1.02–1.49], without heterogeneity [Tau^2^ = 0, *df* = 4, *p* = 0.41, *I*^2^ = 0%].

### Secondary analysis and risk of NDD in offspring

**Fig. 3:**
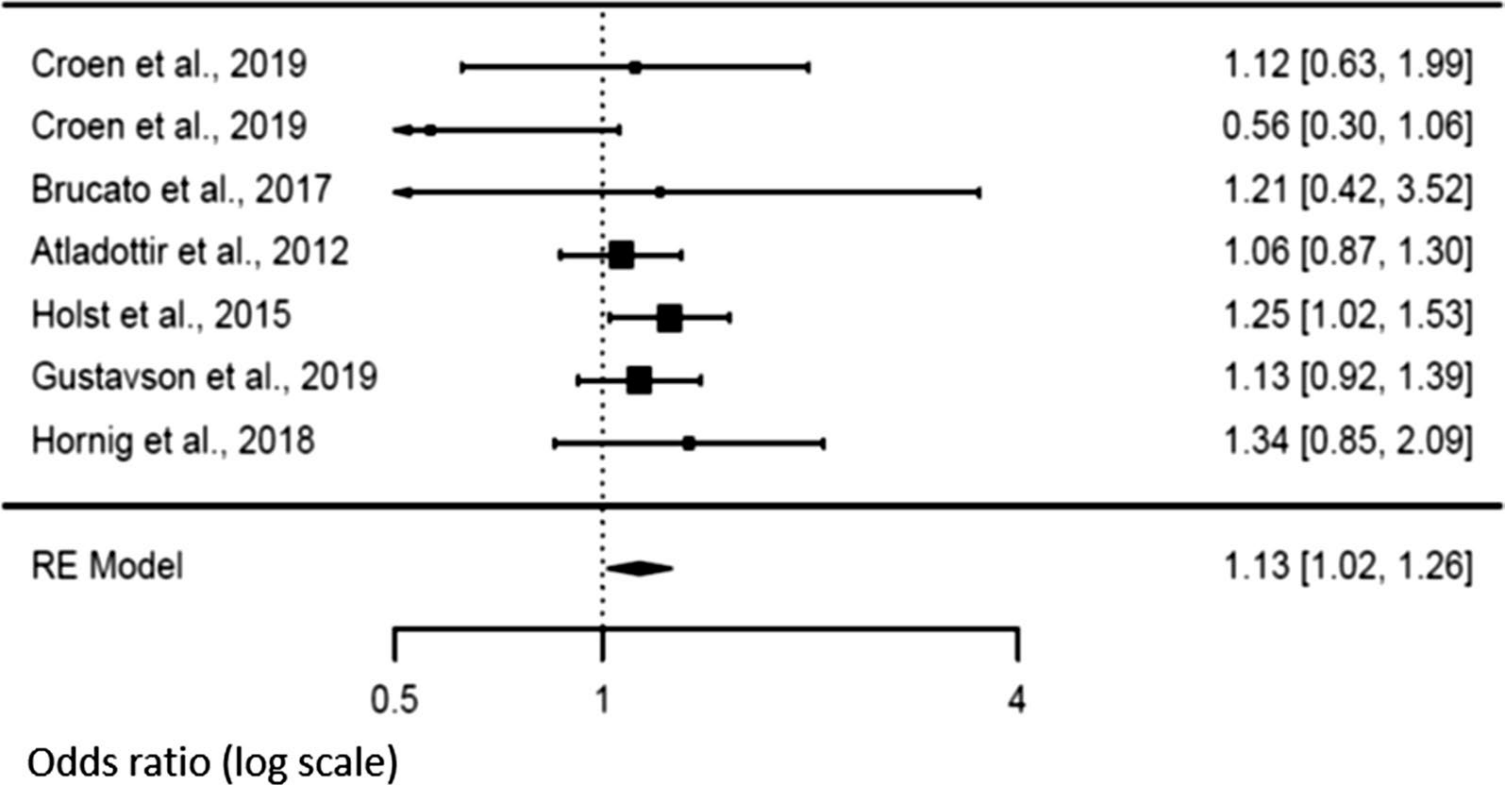
Forest plot relative to fever occurring in the first trimester

**Fig. 4:**
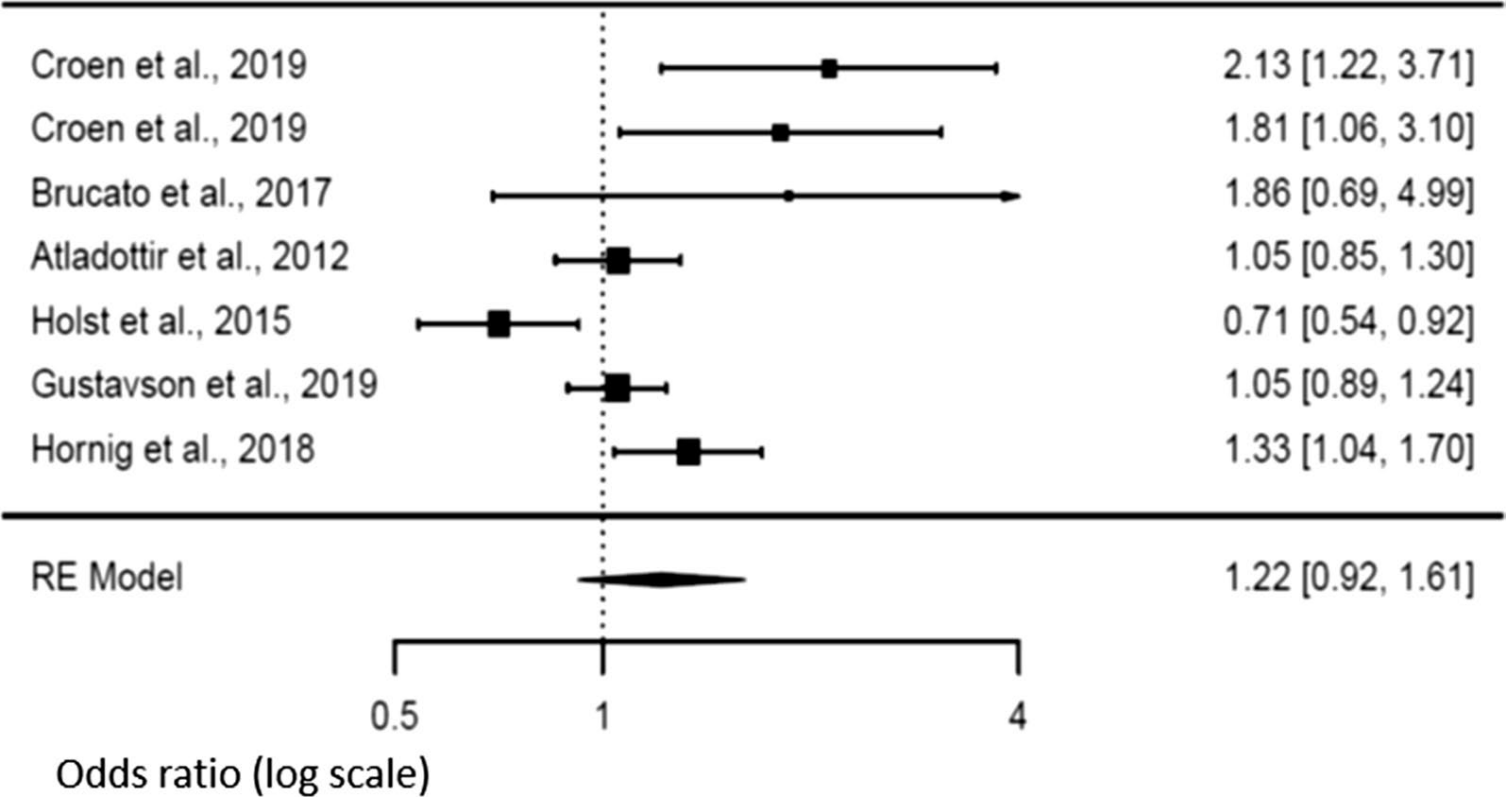
Forest plot relative to fever occurring in the second trimester

**Fig. 5:**
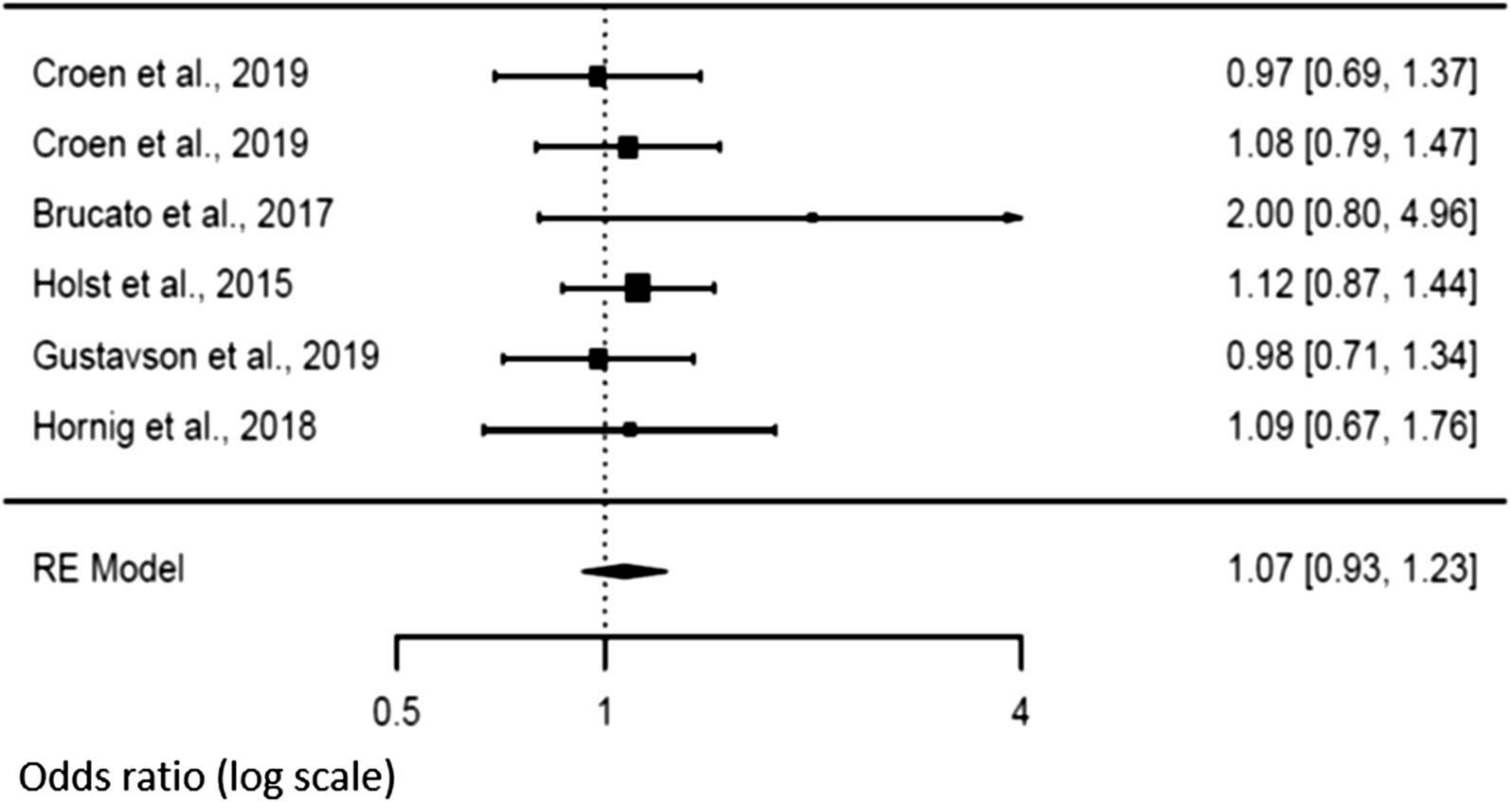
Forest plot relative to fever occurring in the third trimester

An analysis of fever characteristics was conducted to explore sources of heterogeneity in the link between maternal exposure to fever during pregnancy and NDD in the offspring. Six studies [14, 21, 22, 46–48] investigated the effect of maternal fever according to the trimester during which it occurred. Positive association (*p* = 0.024) was noted during the first trimester of pregnancy [OR 1.13; 95% CI 1.02–1.26], without evidence of heterogeneity [Tau^2^ = 0, *df* = 6, *p* = 0.365, *I*^2^ = 0] (Fig. 3). We however did not observe any association between the risk of NDD and the fever occurrence during the second or the third trimesters of pregnancy [OR 1.22 (95% CI 0.92–1.61) and OR 1.07 (95% CI 0.93–1.23) respectively] (Figs. 4 and 5). There was evidence of heterogeneity during the second trimester of pregnancy [Tau^2^ = 0.096, *df* = 6, *p* = 0.0008, *I*^2^ = 82.34], but not during the third trimester [Tau^2^ = 0, *df* = 5, *p* = 0.7741, *I*^2^ = 0]. Funnel plots suggested a potential publication bias for the first and second trimesters, but not for the third one (see Additional file 1: supplemental figures).

**Fig. 6:**
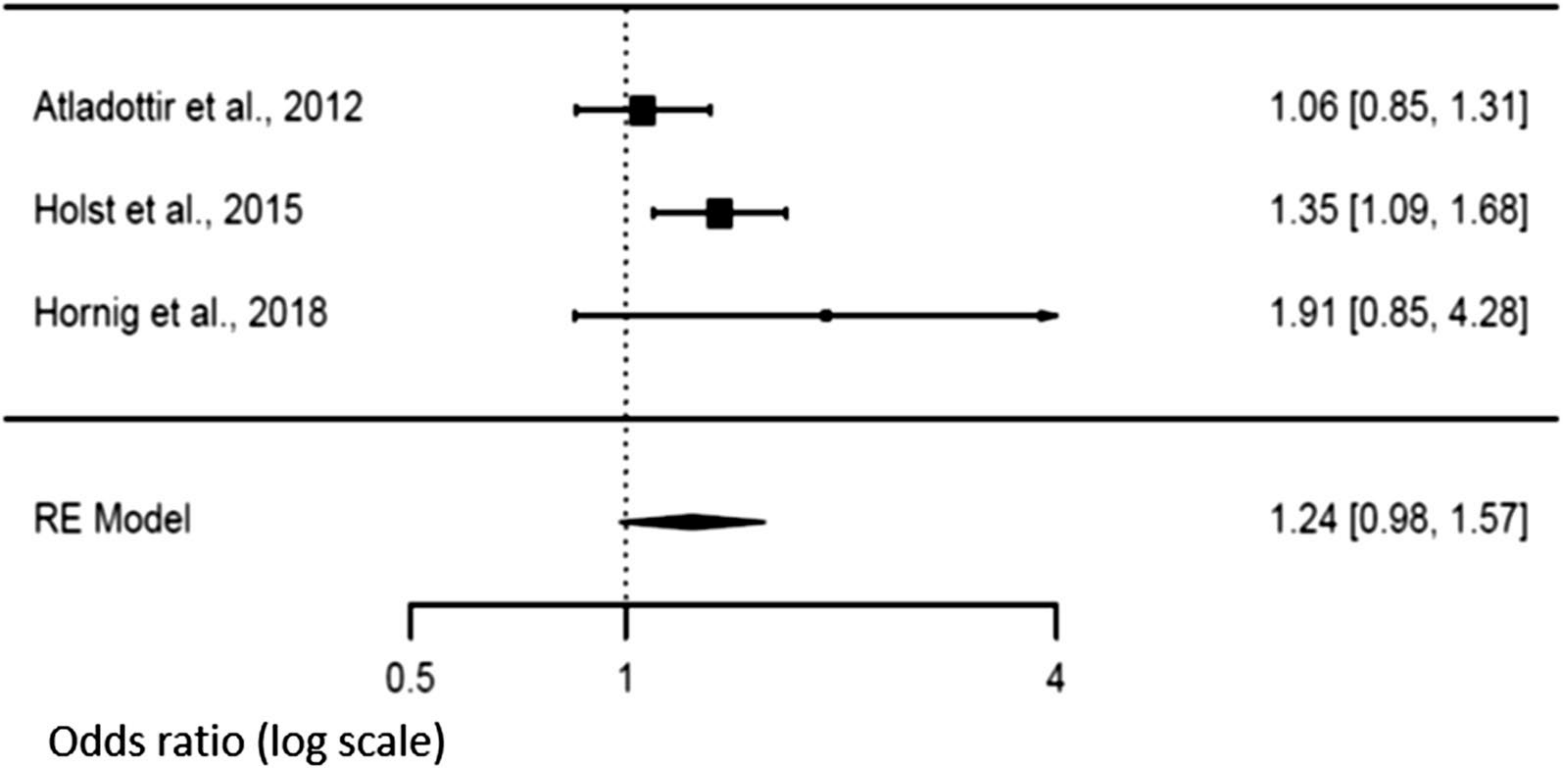
Forest plot relative to fever duration longer than three days

**Fig. 7:**
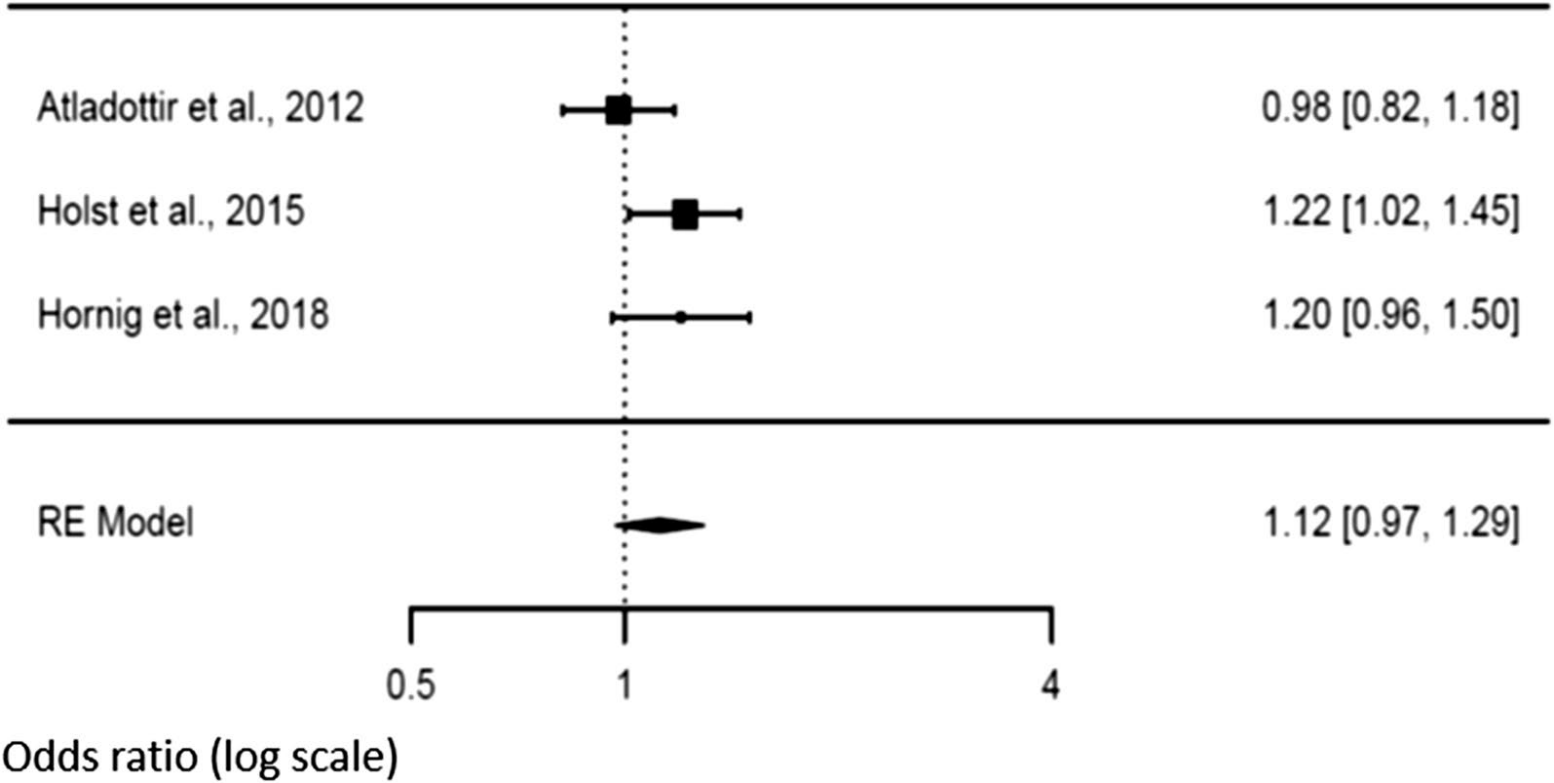
Forest plot relative to fever duration shorter than three days

Three studies [14, 21, 47] explored the association between fever duration and the risk of NDD in the offspring. There was a trend of an increased risk (*p* = 0.072) when fever exceeded 3 days in the mother [OR 1.24 (95% CI 0.98–1.57)] compared to a shorter duration [OR 1.12 (95% CI 0.97–1.29), *p* = 0.1188]. We observed heterogeneity values for a duration longer than 3 days [Tau^2^ = 0.019, *df* = 2, *p* = 0.043, *I*^2^ = 45.91], but not when the studies reported a fever duration shorter than 3 days [Tau^2^ = 0.0064, *df* = 2, *p* = 0.197, *I*^2^ = 39.70]. Funnel plots showed no publication bias (Figs. 6 and 7).

**Fig. 8:**
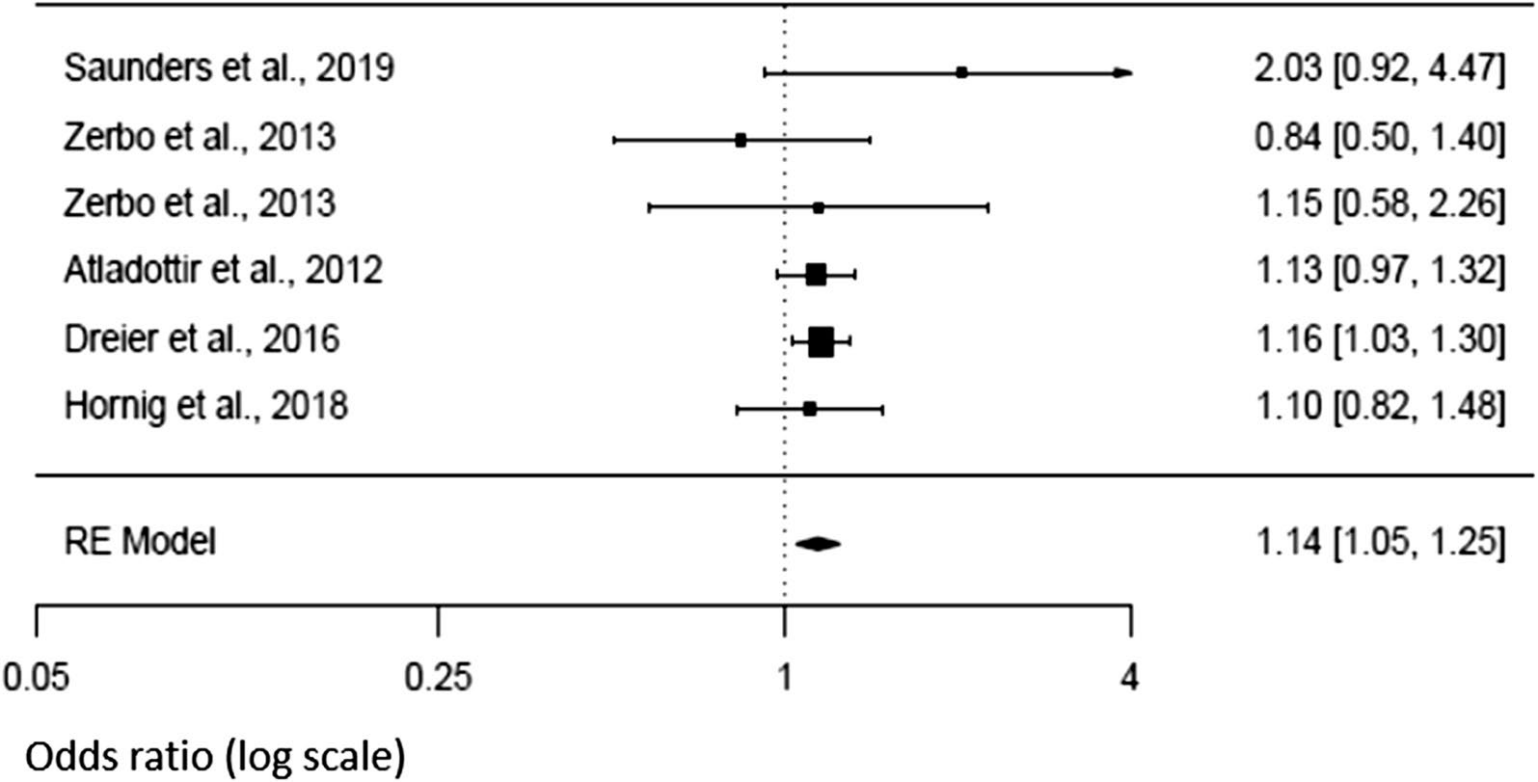
Forest plot relative to fever medication use

Additional secondary analysis suggested that the use of anti-pyretic medication in the context of fever during pregnancy [6, 14, 21, 27, 44] did not significantly affect the risk of NDD in the offspring (*p* = 0.642). There was however evidence for heterogeneity in the studies [Tau^2^ = 0.2462, *df* = 4, *p* < 0.0001, *I*^2^ = 88.24], even in the absence of publication bias (Fig. 8). The other variables we initially selected for the secondary analysis, were not taken into consideration in our results because either they were explored by fewer than three studies, or the metric used to quantify them were not similar among studies.

### Meta-regression: impact of fever duration, antipyretic medication, and maternal characteristics on NDD risk

**Fig. 9:**
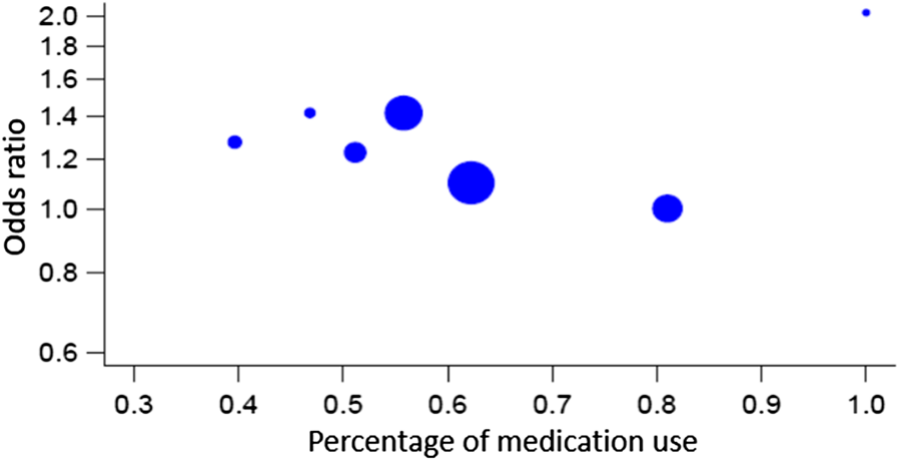
Meta-regression relative to fever medication use

**Fig. 10:**
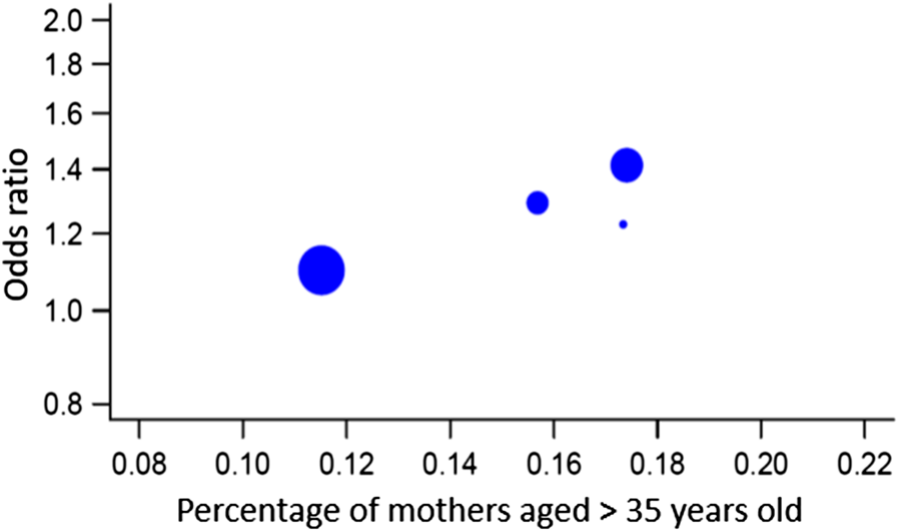
Meta-regression relative to maternal age

To further examine the impact of fever duration (*k* = 3) on the risk of NDD in offspring, we performed a meta-regression. The analysis showed a non-significant effect of prolonged duration (*p* = 0.313), with evidence of heterogeneity [Tau^2^ = 0.0260, *df* = 1, *p* = 0.0164, *I*^2^ = 82.64]. Similarly, the meta-regression model (*k* = 7) for the use of antipyretic medications confirmed the previous findings with no significant association (*p* = 0.264) and evidence of heterogeneity [Tau^2^ = 0.0097, *df* = 5, *p* = 0.0351, *I*^2^ = 54.28] (Fig. 9). We finally explored the effect of maternal age at birth, maternal smoking during pregnancy, and the gestational age at birth. The meta-regression for a maternal age greater than 35 years old (*k* = 4) was a significant covariate (*p* = 0.014), with no heterogeneity [Tau^2^ = 0, *df* = 2, *p* = 0.5142, I^2^ = 0] (Fig. 10). There was no statistically significant association with gestational age at birth over 37 WGA (*k* = 3, *p* = 0.1296) with evidence of heterogeneity [Tau^2^ = 0.0005, *df* = 1, *p* = 0.2991, *I*^2^ = 89.90]. Similarly, no association was observed with maternal smoking during pregnancy (*k* = 4, *p* = 0.6337) with evidence of heterogeneity [Tau^2^ = 0.0132, *df* = 2, *p* = 0.009, *I*^2^ = 75.29].

## Discussion

Our meta-analysis aimed to clarify the relationship between prenatal exposure to fever and neurodevelopmental outcomes in the offspring. Based on a meta-analysis gathering a total of 416,446 pregnant women including 81,124 children who were exposed to maternal fever during pregnancy, we reported an increased risk of NDD in the offspring with an odds-ratio (OR) of 1.24 [95% CI: 1.12–1.38], specifically during the first trimester of pregnancy [OR 1.13; 95% CI 1.02–1.26]. This risk was particularly relevant for ASD (OR 1.15, 95% CI: 1.01–1.31) and DD (OR 1.23, 95% CI: 1.07–1.41).

The increased risk of NDD may directly rise from the increased levels of cytokines and imbalance in T cell populations associated with fever, occurring in a critical window of the fetal brain development [49, 50]. This acute maternal immune dysregulation may affect the in utero environmental homeostasis, required for fetal brain development [49]. Animal models upheld this mechanism, showing a significant deviation in fetal neuron divisions and migrations [17, 51]. Despite infections being the most common route to MIA, fever by itself induced the release of non-specific cytokines, such as Il-6, in pregnant women [14, 52]. It is unclear how peripheral maternal immune signals impacted the brain development [52] but animal studies found that systemic maternal Il-6 secretion was sufficient to induce ASD-like phenotype in the offspring [17]. Added to this, recent studies also suggested that a maternal exposure to stress might induce a downregulation in RNA Binding Motif protein 3 (RBM3) in the developing brain [53]. While RBM3 neuroprotective properties are applied in induced hypothermia, as well as other life-threatening conditions that require cell protection, the mechanisms regulating its expression remained insufficiently covered [53].

Interestingly, we found a significant association between fever during the first trimester of pregnancy and the risk of NDD, in contrast with the last trimester of gestation. Even though the underlying neurobiological hypothesis was not clearly defined, the first two trimesters, specifically between 13 and 27 WGA, entailed an immune profile for appropriate fetal brain growth, characterized by a secretion of Transforming Growth Factor β (TGF-β). The association of fever with increased levels of both Il-6 and TGF-β may directly affect maternal immune cell homeostasis leading to defects in the fetal brain architecture development [20, 54].

Surprisingly, we did not observe that fever duration or anti-pyretic medications during pregnancy influenced the neurodevelopmental risk [6, 14, 21, 27, 44]. These results based on subsamples of subjects may be however unpowered to detect a significant effect on NDD risk. Additionally, we combined antipyretic medications (e.g. acetaminophen, ibuprofen) and antibiotic drugs (only one single study was solely with antibiotics [14]) to explore the whole effect of drugs administrated in the context of fever during pregnancy. A recent meta-analysis found a positive association between acetaminophen and ADHD, with an increased OR of 1.25 (95% CI [1.17, 1.34]) [55]. It is also important to note that recent research suggested that acetaminophen use during third trimester (not only for fever) was associated with ADHD, and ASD onset in the offspring [56].

The meta-regression we ran on maternal confounding factors reported a significant cross-effect for maternal age over 35 years old. This was consistent with previous findings that provided evidence for advanced maternal age as a substantial risk factor for NDD, specifically ASD [7]. We were however unable to detect the cumulative effect of maternal smoking and fever during pregnancy on the risk of NDD. While it was suggested that oxidative stress would trigger the detrimental effects of maternal smoking on fetal brain development, it seemed that maternal fever might follow a distinct pathway [57]. Therefore, these two risk factors might not be closely connected, thus explaining the weak interaction we observed in our results.

Emerging infections drew attention to the impact of fever on the offspring of infected pregnant women [58]. The recent global outbreak of the SARS-CoV-2 raised concerns over the impact of the virus on brain development [58–60]. With still insufficient information on the vertical transmission [61], we need to carefully monitor the developmental trajectory of babies from pregnant women infected by the SARS-CoV-2, specifically in mothers displaying a seroconversion [59].

## Limitations

Although our results were congruent with previous findings in the literature, our meta-analysis should be considered in light of its limitations. First, the inclusion process excluded studies that considered exposure to infections without information on fever. As suggested previously, distinguishing fever outcomes from those of infections might be difficult, giving the fact that both conditions could enhance MIA [14, 26]. Fever as a symptom might not be always present, letting an underlying infection go undetected. Conversely, non-infectious fever is also common during pregnancy but with significant effect on maternal immune regulation [51]. Second, the methodological heterogeneity between cohorts and case–control studies we assumed in our study, may be considered as a publication bias. However, stratification analysis did not support this hypothesis. Similarly, in the subgroup analysis, confounding analysis was limited by the absence of overlapping data between the different studies, especially concerning etiology and administered treatment for fever. Third, we combined the effect of symptomatic treatment for fever and the use of antibiotics under the denomination of antipyretic treatment. While the use of both types of treatment has either a direct or an indirect effect on decreasing the fever, they have different mechanisms that could be linked to the risk of NDD in offspring [14]. Finally, the majority of our population comes from two sources, namely the Danish and the Norwegian cohorts; this could limit generalizability and possibly bias our results. Further studies should take into consideration all that preceded in order to optimize their results.

## Conclusion

In conclusion, our results provided further evidence for an association between maternal fever during pregnancy and NDD risk in the offspring. In line with our results, and in the absence of consensus, it is wise to treat maternal fever accordingly, in order to prevent the previously described subsequent neuroinflammation [28]. In the specific context of SARS-CoV-2 pandemic, careful monitoring for children born from mothers with a febrile episode during pregnancy, specifically during the first trimester and with a seroconversion should be considered [59].

## Supplementary Information

**Additional file 1**. Full data extracted and funnel plots.

## Data Availability

Not applicable.
