# Supplementary material for: Fever during pregnancy as a risk factor for neurodevelopmental disorders: results from a systematic review and meta-analysis
Source: Mol Autism. 2021 Sep 18;12:60. doi: 10.1186/s13229-021-00464-4 (PMC8449704; doi:10.1186/s13229-021-00464-4)
Supplement: Supplementary file 1 — Additional file 1. Full data extracted and funnel plots. [file 13229_2021_464_MOESM1_ESM.docx]

| Author | Year | Location | Study type | NDD | Diagnostic Instrument | Total mean age  (months) | Total Lower age  (months) | Total Upper age  (months) | NDD mean age  (months) | NDD Lower age  (months) | NDD Upper age  (months) | Non NDD Mean age  (months) |
| --- | --- | --- | --- | --- | --- | --- | --- | --- | --- | --- | --- | --- |
| Atladóttir et al. (14) | 2012 | Denmark | Cohort | ASD | ICD10 | NR | 96 | 168 | NR | 124.52 | 141 | NR |
| Dreier et al. (6) | 2016 | Denmark | Cohort | ADHD | ICD10  DSM-IV | NR | 48 | 127.2 | NR | 86.63 | 97.78 | NR |
| Holst et al. (50) | 2015 | Denmark | Cohort | DCD | DCDQ07 | 85 | 84 | 107 | NR | NR | NR | NR |
| Gustavson et al. (51) | 2019 | Norway | Cohort | ADHD | ICD10  ADHD-RS | 136.8 | 88.8 | 207.6 | 132 | 84 | 204 | NR |
| Hornig et al. (21) | 2018 | Norway | Cohort | ASD | ICD10  DSM-IV | 118.64 | 67.2 | 182.4 | 130.08 | NR | NR | 118.57 |
| Saunders et al. (47) | 2019 | Canada | Case Control | ASD | NR | NR | 5 | 120 | NR | NR | NR | NR |
| Croen et al. (22) | 2019 | USA | Case Control | ASD | ADI-R  ADOS-2 | NR | 24 | 60 | NR | NR | NR | NR |
|  |  |  |  | DD | MSEL | NR | 24 | 60 | NR | NR | NR | NR |
| Zerbo et al. (27) | 2013 | USA | Case Control | ASD | ADI-R  ADOS-2 | NR | 24 | 60 | NR | NR | NR | NR |
|  |  |  |  | DD | MSEL | NR | 24 | 60 | NR | NR | NR | NR |
| Christian et al. (48) | 2018 | Jamaica | Case Control | ASD | ADI-R  ADOS-2 | NR | 24 | 96 | NR | NR | NR | NR |
| Brucato et al. (49) | 2017 | USA | Case Control | ASD | ICD9 | NR | NR | NR | NR | NR | NR | NR |

| Author | Non NDD Lower age  (months) | Non NDD Upper age  (months) | Cohort | Fever | Fever NDD | Fever non NDD | No Fever | No Fever NDD | No Fever non NDD | Female Fever | Male Fever | Female No Fever |
| --- | --- | --- | --- | --- | --- | --- | --- | --- | --- | --- | --- | --- |
| Atladóttir et al. (14) | 122,68 | 138,77 | 96736 | 23128 | 234 | 22894 | 61482 | 742 | 72866 | NR | NR | NR |
| Dreier et al. (6) | NR | NR | 89146 | 24531 | 653 | 23878 | 64426 | 1562 | 62864 | NR | NR | NR |
| Holst et al. (50) | NR | NR | 29568 | 7909 | 289 | 7620 | 21603 | 616 | 20987 | 3887 | 4078 | 10564 |
| Gustavson et al. (51) | NR | NR | 99947 | 9100 | 350 | 8750 | 74572 | 2052 | 72520 | 4496 | 4604 | 36373 |
| Hornig et al. (21) | NR | NR | 95754 | 15701 | 113 | 15588 | 80053 | 470 | 79583 | 7671 | 8030 | 38886 |
| Saunders et al. (47) | NR | NR | NR | NR | 20 | 11 | NR | NR | NR | NR | NR | NR |
| Croen et al. (22) | NR | NR | NR | NR | 105 | 124 | NR | NR | NR | NR | NR | NR |
|  | NR | NR | NR | NR | 138 | 124 | NR | NR | NR | NR | NR | NR |
| Zerbo et al. (27) | NR | NR | NR | NR | 97 | 62 | NR | NR | NR | NR | NR | NR |
|  | NR | NR | NR | NR | 32 | 62 | NR | NR | NR | NR | NR | NR |
| Christian et al. (48) | NR | NR | NR | NR | 51 | 22 | NR | NR | NR | NR | NR | NR |
| Brucato et al. (49) | NR | NR | NR | NR | 15 | 78 | NR | NR | NR | NR | NR | NR |

| Author | Male No Fever | Total NDD | Female NDD | Male NDD | Total non NDD | Female non NDD | Male non NDD | Fever T1 | Fever T1 NDD | Fever T1 non NDD | No Fever T1 NDD | No Fever T1 non NDD |
| --- | --- | --- | --- | --- | --- | --- | --- | --- | --- | --- | --- | --- |
| Atladóttir et al. (14) | NR | 976 | 184 | 792 | 95760 | 46952 | 48808 | 10267 | 110 | 10157 | 737 | 72405 |
| Dreier et al. (6) | NR | 2215 | NR | NR | 86742 | NR | NR | NR | NR | NR | NR | NR |
| Holst et al. (50) | 11039 | 905 | NR | NR | 28607 | NR | NR | 3058 | 114 | 2944 | 791 | 25552 |
| Gustavson et al. (51) | 38199 | 2941 | NR | NR | 81270 | NR | NR | 3122 | 99 | 3023 | 2226 | 76916 |
| Hornig et al. (21) | 41167 | 583 | 96 | 487 | 95171 | 46461 | 48710 | 2531 | 20 | 2511 | 454 | 76124 |
| Saunders et al. (47) | NR | 107 | 28 | 79 | 108 | 33 | 75 | NR | NR | NR | NR | NR |
| Croen et al. (22) | NR | 606 | 110 | 496 | 796 | 379 | 417 | NR | 22 | 26 | 584 | 770 |
|  | NR | 856 | 287 | 569 | 796 | 379 | 417 | NR | 16 | 26 | 840 | 770 |
| Zerbo et al. (27) | NR | 538 | 79 | 459 | 421 | 94 | 327 | NR | NR | NR | NR | NR |
|  | NR | 163 | 57 | 106 | 421 | 94 | 327 | NR | NR | NR | NR | NR |
| Christian et al. (48) | NR | 298 | 52 | 246 | 298 | 52 | 246 | NR | NR | NR | NR | NR |
| Brucato et al. (49) | NR | 116 | 31 | 85 | 988 | 585 | 403 | NR | 4 | 29 | 97 | 852 |

| Author | Fever T2 | Fever T2 NDD | Fever T2 non NDD | No Fever T2 NDD | No Fever T2 non NDD | Fever T3 | Fever T3 NDD | Fever T3 non NDD | No Fever T3 NDD | No Fever T3 non NDD | Fever < 3 | Fever < 3 NDD |
| --- | --- | --- | --- | --- | --- | --- | --- | --- | --- | --- | --- | --- |
| Atladóttir et al. (14) | 9488 | 101 | 9387 | 748 | 73186 | NR | NR | NR | NR | NR | 14168 | 140 |
| Dreier et al. (6) | NR | NR | NR | NR | NR | NR | NR | NR | NR | NR | NR | NR |
| Holst et al. (50) | 2617 | 59 | 2558 | 846 | 25938 | 1999 | 68 | 1931 | 837 | 26565 | 4498 | 155 |
| Gustavson et al. (51) | 5352 | 158 | 5194 | 2167 | 74745 | 1444 | 40 | 1404 | 2285 | 78535 | NR | NR |
| Hornig et al. (21) | 9945 | 76 | 9869 | 398 | 68766 | 2623 | 17 | 2606 | 457 | 76029 | 13068 | 92 |
| Saunders et al. (47) | NR | NR | NR | NR | NR | NR | NR | NR | NR | NR | NR | NR |
| Croen et al. (22) | NR | 33 | 21 | 573 | 775 | NR | 64 | 86 | 542 | 710 | NR | NR |
|  | NR | 40 | 21 | 816 | 775 | NR | 99 | 86 | 757 | 710 | NR | NR |
| Zerbo et al. (27) | NR | NR | NR | NR | NR | NR | NR | NR | NR | NR | NR | NR |
|  | NR | NR | NR | NR | NR | NR | NR | NR | NR | NR | NR | NR |
| Christian et al. (48) | NR | NR | NR | NR | NR | NR | NR | NR | NR | NR | NR | NR |
| Brucato et al. (49) | NR | 5 | 24 | 96 | 857 | NR | 6 | 27 | 95 | 854 | NR | NR |

| Author | Fever < 3 non NDD | Fever ≥ 3 | Fever ≥ 3 NDD | Fever ≥ 3 non NDD | Maternal age NDD < 35 | Maternal age non NDD < 35 | Maternal age Fever < 35 | Maternal age No Fever < 35 | Maternal age NDD ≥ 35 | Maternal age non NDD ≥ 35 | Maternal age Fever ≥ 35 | Maternal age No Fever ≥ 35 |
| --- | --- | --- | --- | --- | --- | --- | --- | --- | --- | --- | --- | --- |
| Atladóttir et al. (14) | 14028 | 8730 | 93 | 8637 | 821 | 81242 | NR | NR | 155 | 14518 | NR | NR |
| Dreier et al. (6) | NR | NR | NR | NR | NR | NR | 21831 | 56880 | NR | NR | 2700 | 7546 |
| Holst et al. (50) | 4343 | 2617 | 100 | 2517 | NR | NR | 6762 | 18168 | NR | NR | 1203 | 3435 |
| Gustavson et al. (51) | NR | NR | NR | NR | NR | NR | 7587 | 61520 | NR | NR | 1513 | 13052 |
| Hornig et al. (21) | 12976 | 539 | 6 | 533 | 483 | 78669 | 13049 | 66103 | 100 | 16502 | 2652 | 13950 |
| Saunders et al. (47) | NR | NR | NR | NR | NR | NR | NR | NR | NR | NR | NR | NR |
| Croen et al. (22) | NR | NR | NR | NR | 474 | 607 | NR | NR | 132 | 189 | NR | NR |
|  | NR | NR | NR | NR | 645 | 607 | NR | NR | 211 | 189 | NR | NR |
| Zerbo et al. (27) | NR | NR | NR | NR | 389 | 321 | NR | NR | 149 | 100 | NR | NR |
|  | NR | NR | NR | NR | 120 | 321 | NR | NR | 43 | 100 | NR | NR |
| Christian et al. (48) | NR | NR | NR | NR | 242 | 262 | NR | NR | 56 | 36 | NR | NR |
| Brucato et al. (49) | NR | NR | NR | NR | NR | NR | NR | NR | NR | NR | NR | NR |

| Author | Mean maternal age NDD | Mean maternal age non NDD | Mean WGA NDD | Mean WGA non NDD | Vitamins NDD | Vitamins non NDD | Parity NDD 1 | Parity non NDD 1 | Parity NDD ≥ 2 | Parity non NDD ≥ 2 |
| --- | --- | --- | --- | --- | --- | --- | --- | --- | --- | --- |
| Atladóttir et al. (14) | NR | NR | NR | NR | NR | NR | NR | NR | NR | NR |
| Dreier et al. (6) | NR | NR | NR | NR | NR | NR | NR | NR | NR | NR |
| Holst et al. (50) | NR | NR | NR | NR | NR | NR | NR | NR | NR | NR |
| Gustavson et al. (51) | NR | NR | NR | NR | NR | NR | NR | NR | NR | NR |
| Hornig et al. (21) | NR | NR | NR | NR | NR | NR | NR | NR | NR | NR |
| Saunders et al. (47) | 27,53 | 28,13 | 38,41 | 37,95 | NR | NR | NR | NR | NR | NR |
| Croen et al. (22) | NR | NR | NR | NR | NR | NR | NR | NR | NR | NR |
|  | NR | NR | NR | NR | NR | NR | NR | NR | NR | NR |
| Zerbo et al. (27) | NR | NR | NR | NR | 263 | 236 | 241 | 177 | 297 | 244 |
|  | NR | NR | NR | NR | 75 | 236 | 59 | 177 | 104 | 244 |
| Christian et al. (48) | NR | NR | NR | NR | NR | NR | NR | NR | NR | NR |
| Brucato et al. (49) | 30,11 | 28,25 | 36,5 | 38,4 | NR | NR | NR | NR | NR | NR |

| Author | WGA NDD < 37 | WGA non NDD < 37 | WGA Fever < 37 | WGA No Fever < 37 | WGA NDD ≥ 37 | WGA non NDD ≥ 37 | WGA Fever ≥ 37 | WGA No Fever ≥ 37 | Maternal psychiatric conditions NDD | Maternal psychiatric conditions non NDD |
| --- | --- | --- | --- | --- | --- | --- | --- | --- | --- | --- |
| Atladóttir et al. (14) | 56 | 5971 | NR | NR | 916 | 89497 | NR | NR | 77 | 3688 |
| Dreier et al. (6) | NR | NR | 969 | 2938 | NR | NR | 23562 | 61488 | NR | NR |
| Holst et al. (50) | NR | NR | 271 | 756 | NR | NR | 7702 | 20847 | NR | NR |
| Gustavson et al. (51) | NR | NR | NR | NR | NR | NR | NR | NR | NR | NR |
| Hornig et al. (21) | 23 | 2529 | 431 | 2121 | 560 | 92642 | 15270 | 77932 | NR | NR |
| Saunders et al. (47) | NR | NR | NR | NR | NR | NR | NR | NR | NR | NR |
| Croen et al. (22) | NR | NR | NR | NR | NR | NR | NR | NR | 256 | 247 |
|  | NR | NR | NR | NR | NR | NR | NR | NR | 344 | 247 |
| Zerbo et al. (27) | NR | NR | NR | NR | NR | NR | NR | NR | NR | NR |
|  | NR | NR | NR | NR | NR | NR | NR | NR | NR | NR |
| Christian et al. (48) | NR | NR | NR | NR | NR | NR | NR | NR | NR | NR |
| Brucato et al. (49) | NR | NR | NR | NR | NR | NR | NR | NR | NR | NR |

| Author | Maternal psychiatric conditions Fever | Maternal psychiatric conditions No Fever | Medication NDD | Medication non NDD | Medication Fever NDD | Medication no Fever NDD | Medication Fever non NDD | Medication No Fever non NDD |
| --- | --- | --- | --- | --- | --- | --- | --- | --- |
| Atladóttir et al. (14) | NR | NR | 707 | 69752 | 214 | 493 | 18519 | 51233 |
| Dreier et al. (6) | 2085 | 4574 | 1225 | 42652 | 357 | 868 | 12384 | 30268 |
| Holst et al. (50) | NR | NR | NR | NR | NR | NR | NR | NR |
| Gustavson et al. (51) | 1101 | 6820 | NR | NR | NR | NR | NR | NR |
| Hornig et al. (21) | NR | NR | NR | NR | 50 | NR | 7671 | NR |
| Saunders et al. (47) | NR | NR | 60 | 39 | 20 | NR | 31 | NR |
| Croen et al. (22) | NR | NR | NR | NR | NR | NR | NR | NR |
|  | NR | NR | NR | NR | NR | NR | NR | NR |
| Zerbo et al. (27) | NR | NR | NR | NR | 32 | NR | 63 | NR |
|  | NR | NR | NR | NR | 13 | NR | 44 | NR |
| Christian et al. (48) | NR | NR | NR | NR | NR | NR | NR | NR |
| Brucato et al. (49) | NR | NR | NR | NR | NR | NR | NR | NR |

| Author | No medication NDD | No medication non NDD | No medication Fever NDD | No medication Fever non NDD | No medication No Fever NDD | No medication No Fever non NDD | Medication Fever | Medication No Fever |
| --- | --- | --- | --- | --- | --- | --- | --- | --- |
| Atladóttir et al. (14) | 269 | 13882 | 20 | 4375 | 249 | 9507 | 18733 | 51726 |
| Dreier et al. (6) | 827 | 38320 | 182 | 7553 | 645 | 30767 | 12741 | 31136 |
| Holst et al. (50) | NR | NR | NR | NR | NR | NR | NR | NR |
| Gustavson et al. (51) | NR | NR | NR | NR | NR | NR | 5075 | NR |
| Hornig et al. (21) | NR | NR | 63 | 7315 | NR | NR | 7721 | NR |
| Saunders et al. (47) | 44 | 68 | NR | NR | NR | NR | 31 | NR |
| Croen et al. (22) | NR | NR | NR | NR | NR | NR | NR | NR |
|  | NR | NR | NR | NR | NR | NR | NR | NR |
| Zerbo et al. (27) | 506 | 390 | NR | NR | NR | NR | 63 | NR |
|  | 150 | 390 | NR | NR | NR | NR | 44 | NR |
| Christian et al. (48) | NR | NR | NR | NR | NR | NR | NR | NR |
| Brucato et al. (49) | NR | NR | NR | NR | NR | NR | NR | NR |

| Author | No medication Fever | No medication No Fever | Smoking NDD | Smoking non NDD | Smoking Fever | Smoking No Fever |
| --- | --- | --- | --- | --- | --- | --- |
| Atladóttir et al. (14) | 4395 | 9756 | 205 | 16258 | NR | NR |
| Dreier et al. (6) | 7735 | 31412 | NR | NR | 4147 | 10040 |
| Holst et al. (50) | NR | NR | NR | NR | 1975 | 4904 |
| Gustavson et al. (51) | 4025 | NR | NR | NR | 839 | 5463 |
| Hornig et al. (21) | 7378 | NR | 89 | 8876 | 1490 | 7475 |
| Saunders et al. (47) | NR | NR | 43 | 15 | NR | NR |
| Croen et al. (22) | NR | NR | NR | NR | NR | NR |
|  | NR | NR | NR | NR | NR | NR |
| Zerbo et al. (27) | NR | NR | 66 | 31 | NR | NR |
|  | NR | NR | 11 | 31 | NR | NR |
| Christian et al. (48) | NR | NR | NR | NR | NR | NR |
| Brucato et al. (49) | NR | NR | 24 | 143 | NR | NR |


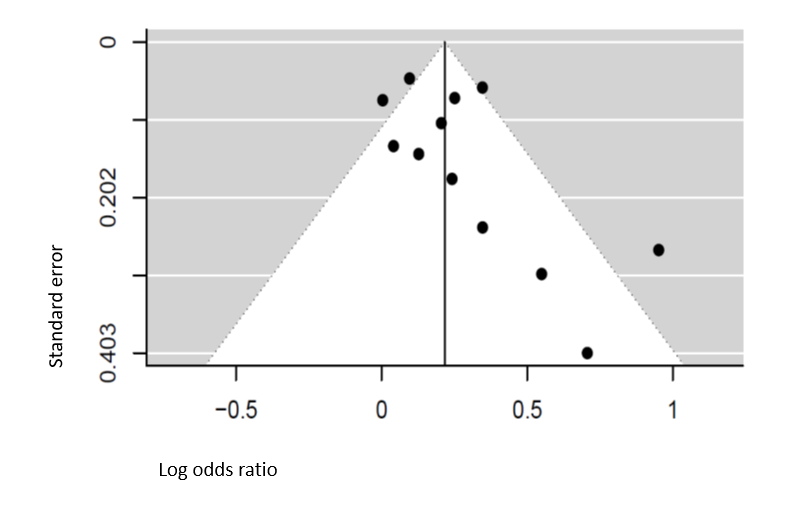
Supplemental figure 1. Funnel plot relative to maternal fever during pregnancy


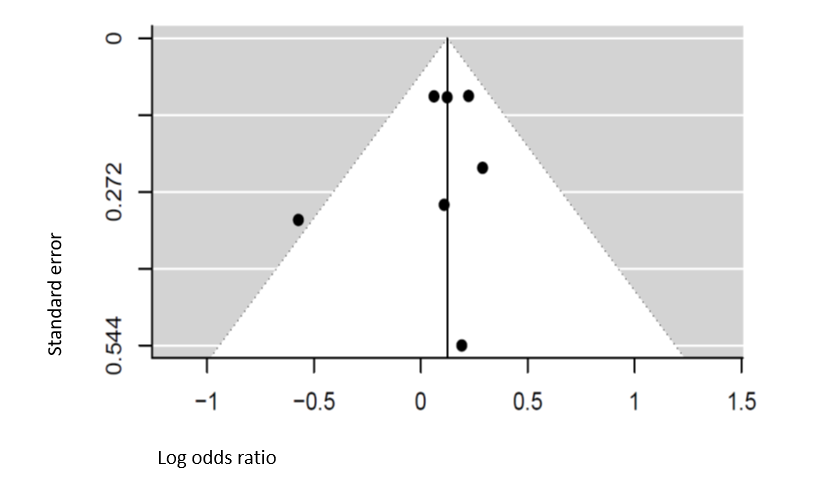
Supplemental figure 2. Funnel plot relative to fever occurring in the first trimester

Supplemental figure3. Funnel plot relative to fever occurring in the second trimester


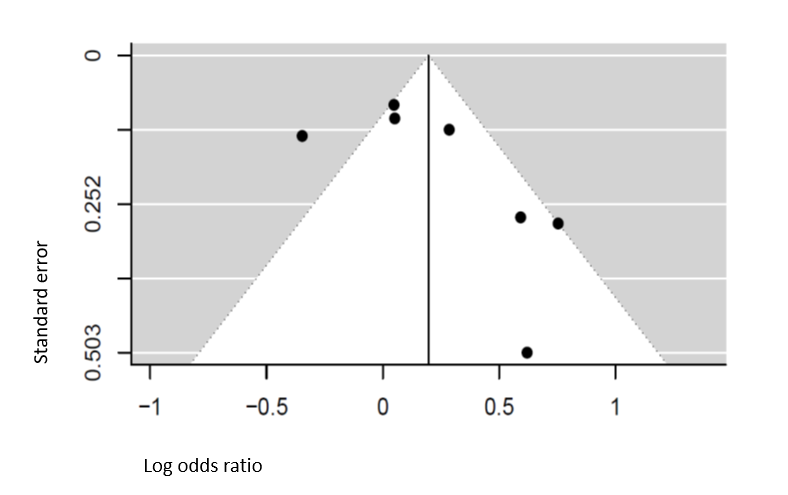


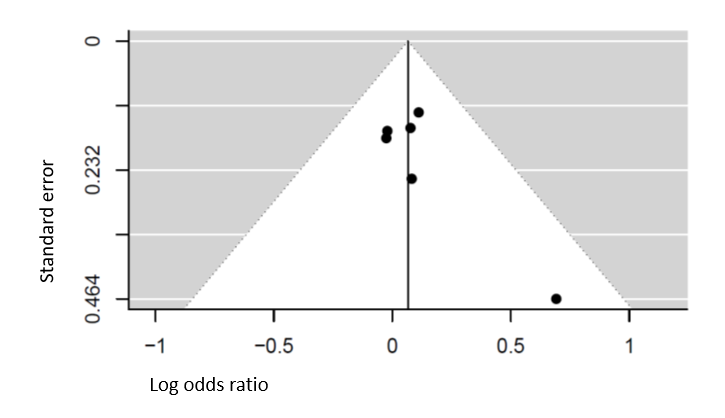
Supplemental figure 4. Funnel plot relative to fever occurring in the third trimester


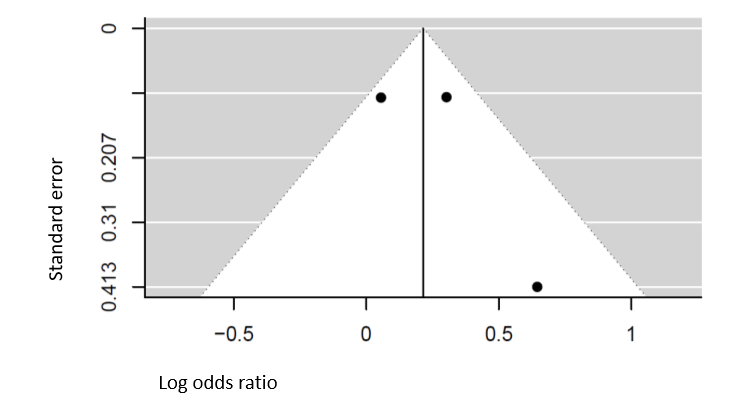
Supplemental figure 5. Funnel plot relative to fever duration longer than three days


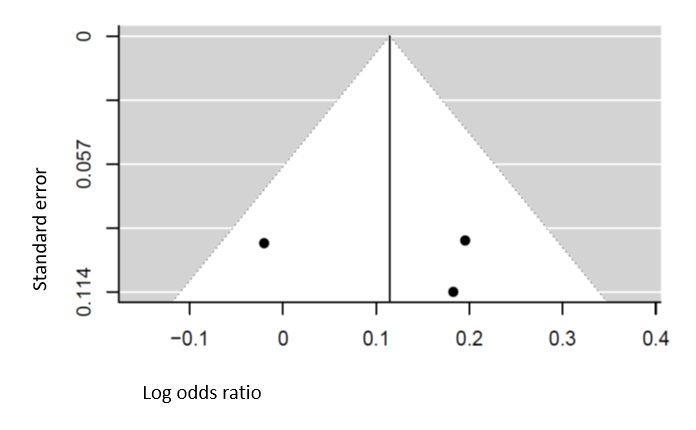
Supplemental figure 6. Funnel plot relative to fever duration shorter than three days


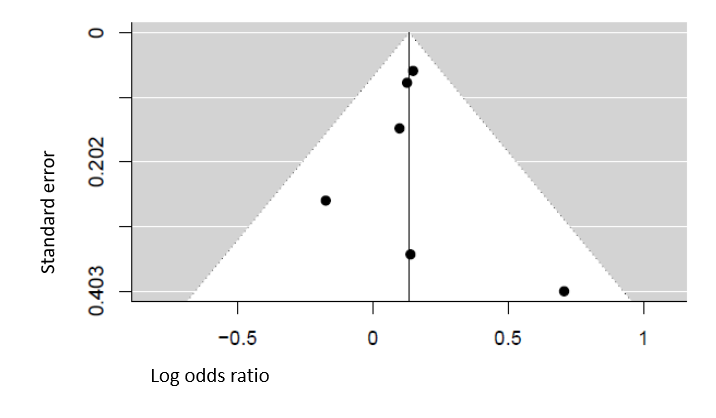
Supplemental figure 7. Funnel plot relative to fever medication use
